# Benign glandular schwannoma

**DOI:** 10.4322/acr.2021.398

**Published:** 2022-09-15

**Authors:** Khaldoon Aljerian

**Affiliations:** 1 King Saud University, College of Medicine, Department of Pathology, Riyadh, Saudi Arabia

**Keywords:** Glandular schwannoma, immunohistochemistry, neurilemmoma, sweat glands neplasms

## Abstract

We report a case of a benign glandular schwannoma in a 63-year-old male who presented with a solitary subcutaneous mass on the left knee, with no previous history of neurofibromatosis type 1. This histological subtype is rare, with only 38 cases reported in the literature. Some of the glands found in this patient resembled sweat glands. These lining stromal spindle cells were positive for S-100 but negative for EMA. S100 was faintly staining the glandular elements. All the glands in the tumor were positive for EMA, particularly at the luminal borders. They were also positive for pancytokeratin. The cystic areas variably show intraluminal, foamy, and hemosiderin-laden macrophages. The different glands expressed two patterns. Some of these were reactive for CK7 and low molecular weight keratin. Immunohistochemical workup is mandatory to assess the neoplastic nature of this glandular component.

## INTRODUCTION

Schwannoma (also known as neurilemmomas) is a benign, encapsulated peripheral nerve sheath neoplasm. It typically presents as a cutaneous schwannoma involving classic areas of Antoni A alternating with hypocellular Antoni B growth patterns and characteristic Verocay bodies. Other variants have also been reported, including plexiform, cellular, epithelioid, glandular, psuedoglandular, and ancient schwannomas.^1^^-^9 Glandular schwannoma is a rare variant characterized by the presence of glands in an otherwise typical schwannoma.^4^ Psuedoglandular schwannoma encompasses gland-like structures (pseudocolumnar epithelial-like cells, loose cuboidal, or epithelial-like cells lacking cellular condensation) or cystic spaces with neoplastic Schwann cell lining.^10^

Histologically, glandular schwannoma comprises spindle cells with serpentine nuclei and demonstrates typical Antoni A (with Verocay bodies) and Antoni B areas and scattered pseudoglandular microcystic foci associated with both areas. In many sections of the glandular schwannoma, mature glandular structures are detected, even if the tumor is not connected to the skin. The lining of the glandular schwannoma is positive for EMA and negative for S100, while the pseudoglandular schwannoma has cystic spaces with no lining, and is negative for EMA but positive for S100. The cystic areas variably show intraluminal, foamy, and hemosiderin-laden macrophages.

This paper highlights one such rare incidence: a case of a benign solitary schwannoma containing two types of benign glands in a patient with no history of neurofibromatosis type 1 or any neural syndromes.

## CASE REPORT

**Figure 1:**
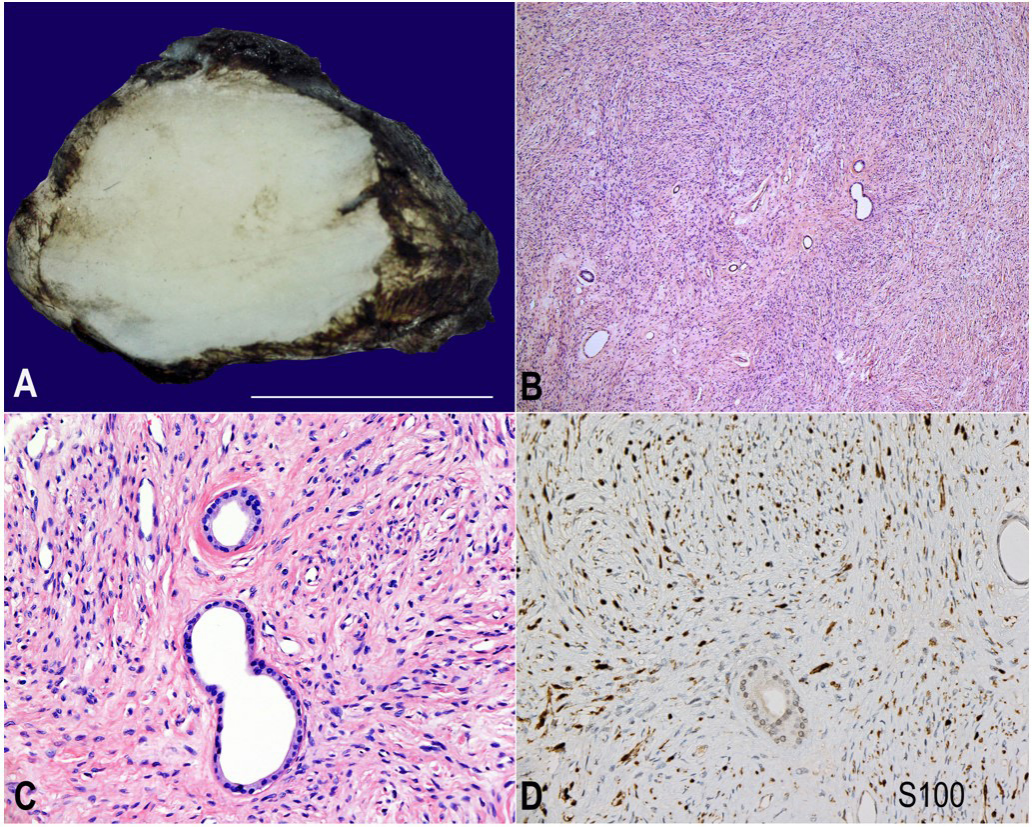
**A –** Cut-section of the 2.5 x 2.0 x 1.0 cm tumor showing an encapsulated, homogenous, tan-whitish tumor, **B, C** and **D –** Photomicrographs of the tumor; **B** – slender background spindle cells and scattered glands (H&E x40); **C –** slender background spindle cells with scattered glands. A myoepithelial cell layer surrounded some glands but not others. The glands appeared to be lined by a single layer of luminal cuboidal cells(H&E x200); **D** – positive S100 immunohistochemical reaction in the background spindle cells. (x100).

A 63-year-old male presented with a subcutaneous soft tissue mass noted on the posterior aspect of the left knee joint. Its size gradually increased for the last two years and remained painless. No neurological deficits were reported. There was no history of neurofibromatosis. No relationship to the large nerve trunk was detected during the mass excision,. The tumor measured 2.5 x 2.0 x 1.0 cm (Figure 1A). It was a sharply demarcated mass almost completely encapsulated by a thin fibrous tissue, had firm consistency, a homogeneous whitish-tan cut surface, and showed no invasive features. No cystic structures were noted. The tumor was mainly composed of interlacing bundles of spindle cells that showed slender, dark-staining, curved, and often wavy nuclei and indistinct cytoplasm. These cells were embedded in a collagenous stroma. Mucoid material within the stroma was sparse. The cells were arranged in fascicles but did not demonstrate nuclear palisading, with minimal Antoni B areas. Neither nuclear pleomorphism nor mitotic figures were detected. In addition, more than 20 glandular structures were found to be scattered throughout the tumor. Deeper cuts into the tumor revealed different areas showing similar scattered glandular elements. The glands were not connected, and they lacked cluster formation. They were lined with cuboidal or columnar cells, with clear and weakly eosinophilic cytoplasm. The glandular epithelium was negative for mucin, as demonstrated by histochemistry (Alcian blue and mucicarmine stains). Some of these glandular cells had a basal cell layer under the luminal layer, composed of smooth muscle cells (Figures 1B and 1C).

**Figure 2:**
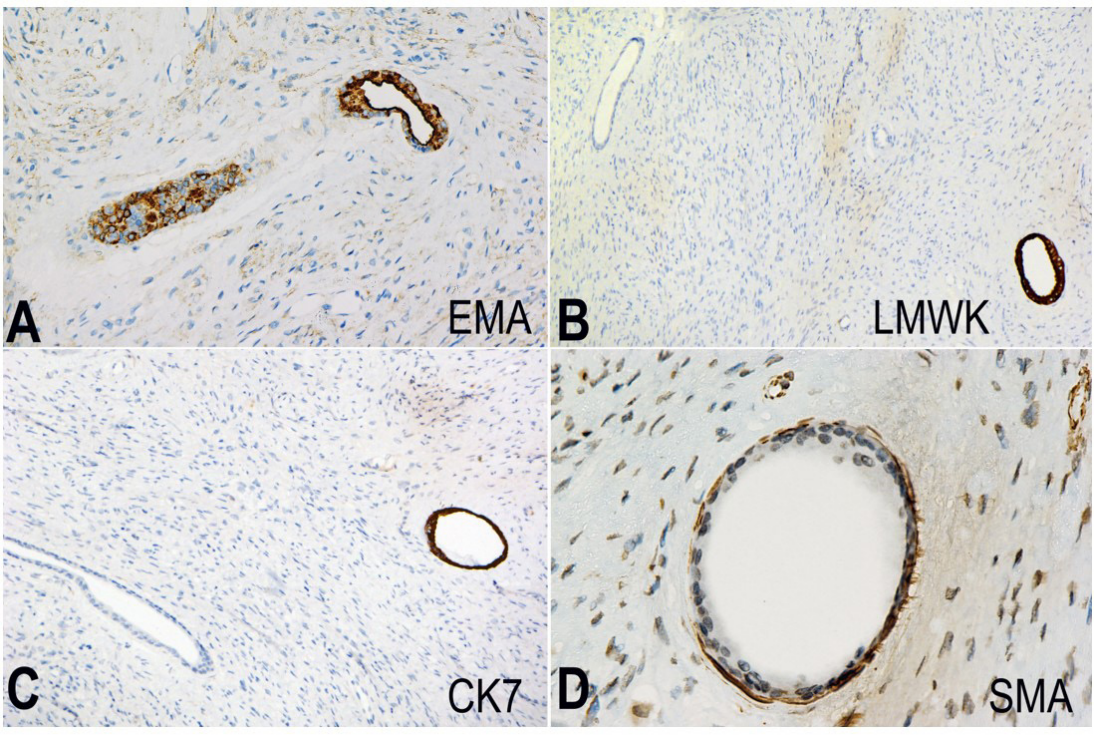
Photomomicrographs of the tumor. **A –** Epithelial membrane antigen (EMA) stain expressed by the epithelial cells. (EMA x200); **B** – The luminal cells of some of the glands expressed the low molecular weight keratin stain (LMWK x100); **C** – The luminal cells of some of the expressed the cytokeratin stain (CK7 x100); **D** – The smooth muscle actin stain was expressed by the myoepithelial cell layer that was present in some of the glands in this neurofibroma. Note: not all the glands were positive for this stain. (SMA x400).

Many stromal spindle cells were diffusely and strongly reactive for the S-100 protein (Figure 1D) but not for EMA (Figure 2A).

S-100 faintly stained the glandular elements. All glands in the tumor were positive for EMA, particularly at the luminal borders. They were also positive for pancytokeratin. The different glands expressed two patterns. Some were reactive to CK7 and low molecular weight keratin (LMWK) (Figures 2B and 2C). The same glands appeared to have a second basal layer positive for smooth muscle actin (SMA) (Figure 2D). The rest of the glands were negative for CK7 and LMWK. CK20, chromogranin A, and synaptophysin were negative in both types of glands.

## DISCUSSION

Glandular elements in neurofibromas that fulfill the criteria developed by Woodruff and Christensen^2^ and Christensen et al.^11^ are scarce findings. Only a few reports have described such occurrences and suggested different hypotheses.^12^^-^14 The glandular elements have been suggested to originate from skin entrapment.^15^ Other potential reasons behind the origin of these glandular structures is metaplasia^11^ or the glands being induced by the tumor from pluripotential neural crest cells rather than being entrapped.^12^^,^^16^ A previously reported glandualr schwannoma, which questioned the distinguishing of true metaplasia from glandular entrapment, was very similar to our reported case^17^.

The differential diagnoses for this case included perineuroma, neurofibromas, and schwannoma-like pleomorphic adenoma. Because of the non-reactivity of the spindle cell component to EMA, the diagnosis of a perineuroma was excluded. The findings above fulfilled two of the original criteria described by Woodruff and Christensen for diagnosing a PNST,^2^ which made neurofibroma a suitable diagnosis for our case. Neurofibromas do not have a sharp border, and entrapment of the sweat glands is common, both of which help to distinguish them from a schwannoma histologically. In this case, the tumor origin of the glandular structures was determined by the immunohistochemical staining of the different glands. The sweat glands consist of two portions – the secretory coil (which was positive for CK7 and contained SMA-positive myoepithelial cells) and ducts (which were negative for CK7 and did not have myoepithelial cells). Therefore, the morphology and immunoprofile (including the S-100 positivity) of the glands in this case matched the typical finding for sweat glands, and eliminated the metaplastic or neoplastic origin of these structures. It is also improbable for any tumor to develop such complex metaplastic components, including two different portions, each with every feature of either the secretory coil or ducts of the sweat glands.^1^^-^5

The immunohistochemical staining pattern of the glands with a myoepithelial cell layer within the tumor was similar to that found in the eccrine glands within an intact dermis. In contrast, other glands that lacked the myoepithelial cell layer, which was negative for SMA, LMWK, and CK7 stains. This diverse and dimorphic pattern of glandularity may prove that these glands were made within this tumor and not entrapped.

## CONCLUSION

Glandular schwannoma is considered a rare variant with divergent differentiation and of considerable interest to pathologists since it must be differentiated from morphologically similar tumors. Immunohistochemical investigations are beneficial for establishing the diagnosis. Our case supports the theory that glandular elements in glandular schwannoma are genuine rather than skin adnexal entrapment.

## References

[1] Chuang ST, Wang HL (2007). An unusual case of glandular schwannoma. Hum Pathol.

[2] Woodruff JM, Christensen WN (1993). Glandular peripheral nerve sheath tumors. Cancer.

[3] Uri AK, Witzleben CL, Raney RB (1984). Electron microscopy of glandular schwannoma. Cancer.

[4] Kim YC, Park HJ, Cinn YW, Vandersteen DP (2001). Benign glandular schwannoma. Br J Dermatol.

[5] Holliday AC, Mazloom SE, Coman GC, Kolodney MS, Chavan RN, Grider DJ (2017). Benign glandular schwannoma with ancient change. Am J Dermatopathol.

[6] Ferry JA, Dickersin GR (1988). Pseudoglandular schwannoma. Am J Clin Pathol.

[7] Deng A, Petrali J, Jaffe D, Sina B, Gaspari A (2005). Benign cutaneous pseudoglandular schwannoma: a case report. Am J Dermatopathol.

[8] Ide F, Obara K, Mishima K, Saito I (2006). Intraparotid pseudoglandular schwannoma. J Oral Pathol Med.

[9] Lisle A, Jokinen C, Argenyi Z (2011). Cutaneous pseudoglandular schwannoma: a case report of an unusual histopathologic variant. Am J Dermatopathol.

[10] Ud Din N, Ahmad Z, Ahmed A (2016). Schwannomas with pseudoglandular elements: clinicopathologic study of 61 cases. Ann Diagn Pathol.

[11] Christensen WN, Strong EW, Bains MS, Woodruff JM (1988). Neuroendocrine differentiation in the glandular peripheral nerve sheath tumor. Pathologic distinction from the biphasic synovial sarcoma with glands. Am J Surg Pathol.

[12] Joshi D, Gangane N, Kishore S, Vagha S (2008). Unusual histological presentation in neurofibromas: two case reports. Cases J.

[13] Kusumi T, Tanaka M, Kurita T (2001). Non-neoplastic glandular structures in a benign peripheral nerve sheath tumor. Pathol Int.

[14] AlAli BM, Amr SS (2021). Malignant glandular triton tumor arising in the radial nerve with prolonged survival: a case report and review of the literature. Case Rep Pathol.

[15] Callagy G, Thornton J, Rawluk D, Farrell MA (2000). Benign glandular peripheral nerve sheath tumor of the seventh and eighth cranial nerve complex. Clin Neuropathol.

[16] Schaefer IM, Agaimy A, Fletcher CDM, Hornick JL (2017). Claudin-4 expression distinguishes SWI/SNF complex-deficient undifferentiated carcinomas from sarcomas. Mod Pathol.

[17] Saggini A, Di Prete M, D’Amico F, Lora V, Orlandi A (2019). Glandular Schwannoma: an uncommon variant of schwannoma with controversial histogenesis. Dermatopathology (Basel).

